# Cordyceps sinensis extract protects against acute kidney injury by inhibiting perforin expression in NK cells via the STING/IRF3 pathway

**DOI:** 10.18632/aging.205676

**Published:** 2024-03-21

**Authors:** Shuang Li, Wei Pang, Yuzhu Wang, Yiting Zhang

**Affiliations:** 1 General Department of Western Medicine, Yangjing Community Health Service Center, Shanghai 200135, China; 2 Department of Emergency Medicine, Xinhua Hospital Affiliated to Shanghai Jiao Tong University School of Medicine, Shanghai 200092, China; 3 General Department of Traditional Chinese Medicine, Yangjing Community Health Service Center, Shanghai 200135, China

**Keywords:** AKI, NK cells, perforin, cordyceps sinensis, 2’-deoxyadenosine

## Abstract

Acute kidney injury (AKI) is associated with immune cell activation and inflammation. However, the putative pathogenic mechanisms of this injury have not been thoroughly investigated. Natural killer (NK) cells play an important role in immune regulation; however, whether NK cells regulate AKI remains unclear. Cordyceps sinensis (CS), a modern Chinese patented medicine preparation, has been widely used in treating patients with chronic kidney disease (CKD) owing to its anti-inflammatory effects and maintenance of immune homeostasis. Whether 2’-deoxyadenosine, a major active component in CS, can ameliorate renal AKI by regulating immunity, particularly in NK cells, has not been reported. This study is the first to demonstrate how NK cells promote AKI by releasing perforin, interferon-gamma (IFN-γ) and other inflammatory factors *in vivo* and *in vitro*. Differential gene expression between AKI and normal tissues was assessed using bioinformatic analyses. Quantitative real-time PCR, western blotting, and immunohistochemical staining were used to detect target protein mRNA and protein expression. Levels of inflammatory factors were measured using enzyme-linked immunosorbent assay. We found the high doses of the 2’-deoxyadenosine treatment significantly alleviated FA-induced renal damage *in vivo*, and alleviated the NK cells of renal injury by activating the STING/IRF3 pathway to inhibit perforin release *in vitro*. The results showed that 2’-deoxyadenosine could mitigate AKI by downregulating the activity of NK cells (by decreasing the expressions of perforin and IFN-γ) and inhibiting the stimulator of interferon genes and phosphorylated IFN regulatory factor 3. This may provide valuable evidence supporting the clinical use of CS in treating patients with AKI.

## INTRODUCTION

Acute kidney injury is a common clinical illness characterized by sudden loss of renal function. This condition can lead to renal fibrosis and end-stage renal failure with a high risk of death. In recent years, AKI has accounted for 67.2% of multiple organ injuries caused by global emergencies and natural disasters [1, 2]. The causes and mechanisms of AKI remain to be understood [3–6]. Increasing evidence has shown that immune cells and cytokines are involved in the regulation of AKI [7–10]. The release of interferon-gamma (IFN-γ) and tumor necrosis factor-alpha (TNF-α) can induce other inflammatory cytokines and responses, which are associated with the damage of renal tubular cells and kidney tissue [11, 12]. Furthermore, IFN-γ produced by activated natural killer cells partially activates the perforin pathway, causing glomerular endothelial cell injury and AKI with hematuria [13]. Stimulator of interferon genes (STING, also known as MITA, MPYS, ERIS, and TMEM173) is an important protein that connects DNA sensors and downstream factors in the innate immune signaling pathway [14]. The second messenger, cyclic adenosine monophosphate (cGAMP), directly activates STING and its key synthase loop, GMP-AMP synthase (cGAS), subsequently recruiting STING to TANK-binding kinase 1 (TBK1) and activating downstream signaling molecules, such as IFN regulator 3 (IRF3), to promote type I IFN gene expression [15–17]. However, the exact mechanism of NK cell activation via the STING/TBK1/IRF3 pathway in AKI has not yet been elucidated.

Cordyceps sinensis, a traditional Chinese medicine, has been used to improve health, especially after prolonged treatment of kidney diseases [18, 19]. Several studies have confirmed that CS plays various roles in inhibiting tumor growth, treating inflammation and oxidative stress, promoting the proliferation of tubular epithelial cells *in vitro*, and accelerating the repair of damaged cells [20, 21]. In addition, CS reduces renal vascular resistance and improves nephrotoxicity-induced renal dysfunction in mice via antioxidant, anti-apoptotic, and anti-autophagic mechanisms [22, 23]. However, the mechanism by which CS repairs acute renal dysfunction and the main components, each active ingredient of CS, have not been thoroughly investigated. 2’-deoxyadenosine and 3’-deoxyadenosine (cordycepin), the major active components of CS, have been used as valuable chemical markers for CS quality control [24]. In this study, we aimed to evaluate the effects of a CS extract comprising 2’-deoxyadenosine on the prevention of AKI.

## MATERIALS AND METHODS

### RNA-seq transcriptome analysis

### Screening and processing of the original sequence data

Using the GEO datasets of the National Center for Biotechnology Information (NCBI; https://www.ncbi.nlm.nih.gov/), datasets GSE145085 and GSE145085 and the biological project PRJNA605973 related to AKI were obtained. Eight public FASTQ files in PRJNA605973 were downloaded: SRR11066831, SRR11066835, SRR11066839, SRR11066843, SRR11066847, SRR11066851, SRR11066855 and SRR11066859. Public FASTQ files were downloaded from the European Nucleotide Archive (https://www.ebi.ac.uk/ena). Trim-galore (v0.6.5-1) was used to trim the original sequence. FastQC and MultiQC were used to obtain gene quality control reports. Raw sequence reads were aligned with the human genome hg19 using Subjunc (v2.0.1). Gene expression levels were quantified using featureCounts (v2.0.1) based on the GENCODE human gene model version 19 (GRCh37.p13), and the expression matrix was obtained.

### Differential gene expression and enrichment analyses

The expression matrix was assessed using intergroup and principal component analyses (PCA). Differential gene expressions between the cisplatin and untreated groups were predicted by a linear model using the Bioconductor package “DESeq2” [25]. Gene ontology (GO) and Kyoto Encyclopedia of Genes and Genomes (KEGG) metabolic pathway enrichment analyses were performed using the Bioconductor package “clusterProfiler” to obtain the key pathway information and other related information, visualized using R language [26].

### Protein-protein interaction (PPI) network analysis

The PPI network was analyzed through the STRING pathway (https://string-db.org).

### Isolation and purification of CS

**Figure 1:**
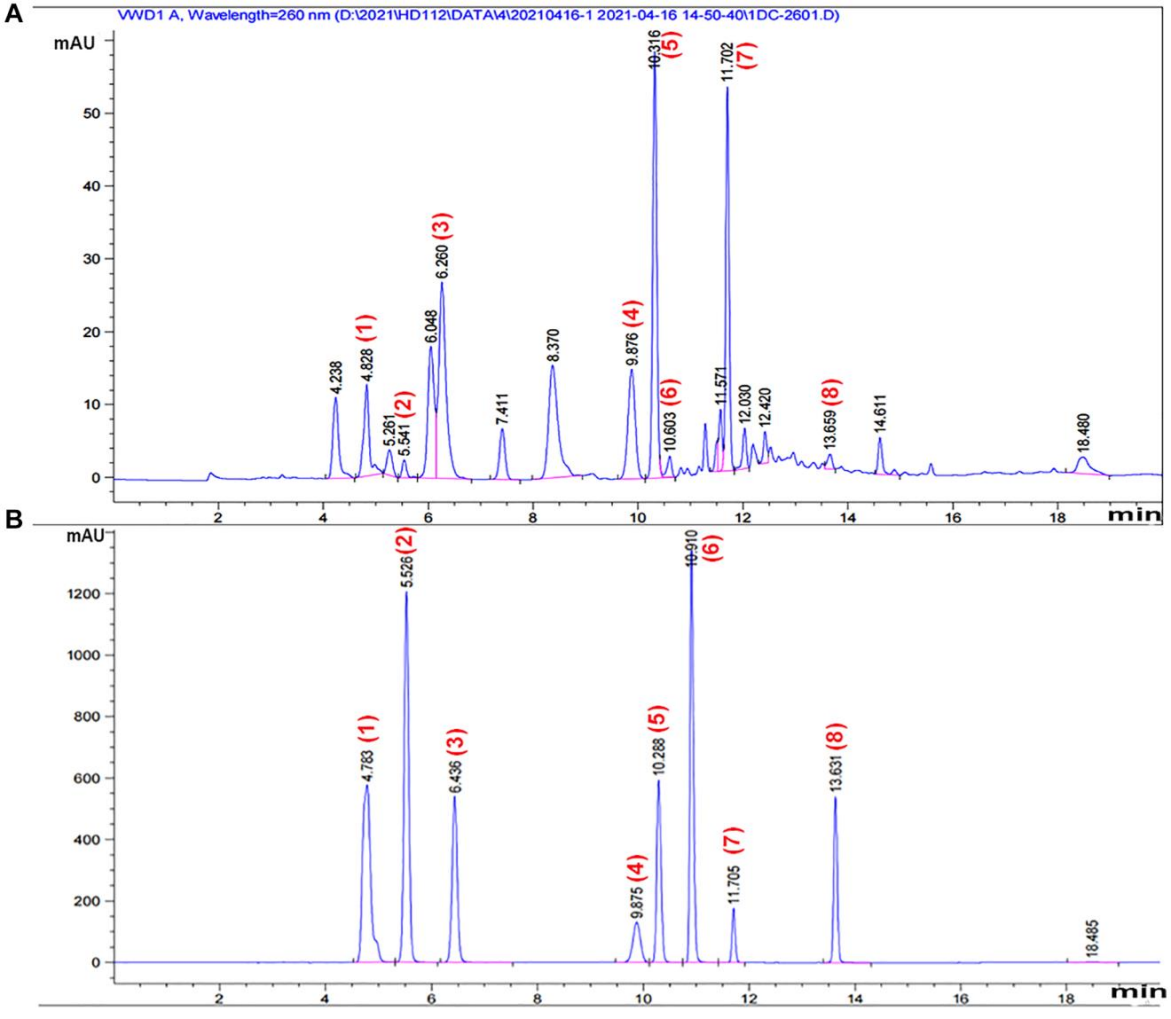
**HPLC chromatogram of CS.** A representative HPLC chromatogram was acquired at 260 nm of (**A**) component-rich extract obtained from CS and (**B**) standards. Peaks were tentatively identified as: 1, adenine 2, uracil; 3, hypoxanthine; 4, uridine; 5, adenosine; 6, 2’-deoxyadenosine; 7, guanosine hydrate; 8, thymidine.

CS (trade name Corbrin Capsule), also known as Bailing Jiaonang in the Chinese Pharmacopoeia (Lot number: 2001103), was obtained from Hangzhou Zhongmei Huadong Pharmaceutical Co., Ltd. (Hangzhou, China). 2’-deoxyadenosine was purchased from MedChemExpress (HY-W040329, USA). CS powder contains a variety of water- and alcohol-soluble nucleoproteins, including proteins, amino acids, mannitol, nucleosides (such as adenosine and cordycepin), nucleobases, sterols, saturated and unsaturated fatty acids, purines, and pyrimidines. Therefore, alcohol, hydrolysis, and enzymatic hydrolysis have been used for CS extraction. Alcohol extraction: CS powder (10 kg) was soaked in 80% medicinal alcohol for 2 days, and the alcohol extract was obtained by filtration and concentration under reduced pressure. Hydrolysis: Ten times the volume of pure water was added to the residue, which was then boiled for 2 h. This step was repeated to obtain the aqueous extract, which was then concentrated under reduced pressure, heated, and concentrated in the upper compartment of an electric furnace. Trypsin was added to the residue after water extraction, and enzymolysis was performed. The supernatant was separated by centrifugation, and the enzyme was eliminated. Subsequently, the three concentrated solutions were combined and homogenized to obtain 9.8 L of bacterial solution (1.02 g/mL), which was sterilized using UV light. Owing to the incomplete extraction process, CS was lost during the operation. If 3% was used to compensate for the loss, the concentration of the CS extract was calculated as 1.05 g/mL. Nucleosides and nucleobases in CS were analyzed using a high-performance liquid chromatography (HPLC) system (Agilent Technologies, Santa Clara, CA, USA) [27]. An ULtimate AQ-C18 (4.6 mm × 250 mm, 5 μm) column was employed to purify nucleosides and nucleobases. The mobile phase was composed of 0.1% (v/v) phosphoric acid solution as eluent A and methanol as eluent B. Gradient elution was conducted as follows: 0–5 min, 0% B; 5–10 min, 20% B; 10–15 min, 50% B; 15–16 min, 0% B. The column temperature was 40°C, the flow rate was 1.0 mL/min, and the injection volume was 5°L. UV/vis spectra were measured over a wavelength range of 200–400 nm, and the peaks of the nucleosides and nucleobases were captured at 260 nm. Among the quantified nucleoside and nucleobase compounds, adenosine was the most abundant in CS (2136.96 g/mL, followed by 2’-deoxyadenosine (203.36 g/mL) (Figure 1) (Tables 1 and 2).

**Table 1:** Peak and area of components of CS using multiplier and dilution factor with ISTDs.

**Compounds**	**RetTime (min)**	**Area (mAU×s)**	**Content (mg/mL)**
Adenine	4.828	93.84496	390.848
Uracil	5.261	25.54213	73.408
Uridine	9.876	144.6443	1342.176
Adenosine	10.316	317.055	2136.96
2’-Deoxyadenosine	10.603	17.71727	203.36
Guanosine hydrate	11.702	234.1245	20.25
Thymidine	13.659	12.78851	1.46

**Table 2:** Peak and area of standards using multiplier and dilution factor with ISTDs.

**Compounds**	**RetTime (min)**	**Area (mAU×s)**
Adenine	4.783	6206.14
Uracil	5.526	7423.013
Hypoxanthine	6.436	3677.84
Uridine	9.875	1316.039
Adenosine	10.288	3366.058
2’-Deosyadenosine	10.91	6287.043
Guanosine hydrate	11.705	745.9741
Thymidine	13.631	2487.645

### Herb formulation ingredient collection and active ingredient-associated target prediction

The TCM Systems Pharmacology Database and Analysis Platform (TCMSP) is a unique platform that provides pharmacological information on traditional Chinese medicine, including herbs. According to the literature recommendations of TCMSP, OB ≥ 30% and DL ≥ 0.18 were selected as screening thresholds. DrugBank data, a network-based tool for predicting the most likely targets of small molecules, was used to screen the relevant targets of each chemical component [28–31].

### Animals and treatments

A total of 30 male C57BL/6 mice (Charles River, Wilmington, MA, USA, approved by the Laboratory Animal Welfare and Ethical Review Board of Shanghai Jiao Tong University School (Number: XHEC-STCSM-2022-051), weighing 25–30 g and aged 8–10 weeks, were randomly and equally divided into five groups (*n* = 6 per group): normal control (NC) group, folic acid (FA) group, and low-dose 2’-deoxyadenosine-treated (L-Deo) group, medium dose 2’-deoxyadenosine-treated (M-Deo) group, and high-dose 2’-deoxyadenosine-treated group (H-Deo). Mice in the FA group were intraperitoneally injected with a single dose of folic acid (100 mg/kg dissolved in 0.9% NaHCO_3_) to induce kidney injury. Renal injury was also induced in the FA + CS group (*n* = 10), similar to that in the FA group. Nevertheless, the mice were immediately treated with CS extract (150 mg/kg), and CS injection lasted for two consecutive days following folic acid injection. Mice in the NC group (*n* = 10) were injected with equal amounts of saline. The mice were then placed on a thermostat to maintain the body temperature at 36.5–37°C and sacrificed 48 h after disposal. The left kidneys of the mice were harvested and snap-frozen for the isolation of RNA or proteins, and the right kidneys were perfused with phosphate-buffered saline (PBS) and fixed in 10% buffered paraffin for histological examination.

### Cells and culture conditions

NK92 cells (ATCC) were stimulated for 4–8 days with or without IL-2 (1000 IU/mL) in RPMI 1640 medium supplemented with 10% fetal calf serum, penicillin (100 U/mL), streptomycin (100 g/mL), glutamine (2 mM), sodium pyruvate (1 mM), HEPES (10 mM), and 2-ME (0.5 mM) under a 5% CO_2_ atmosphere at 37°C. Stimulated NK cells were used as effector cells.

### Biochemical assay and enzyme-linked immunosorbent assay (ELISA)

Mouse blood was collected in a tube containing heparin using the tail-clamping method, and plasma was collected by centrifugation. According to the manufacturer’s protocol, SCr and BUN plasma concentrations were determined using kits from the Nanjing Jiancheng Institute of Biological Engineering (C011-2-1, C013-2-1). Kidney injury molecule-1 (Kim-1) and neutrophil gelatinase-associated lipocalin (NGAL) levels were measured using an ELISA Kit (Abcam, ab213477, ab199083, USA).

### Histology and immunohistochemistry (IHC)

Mouse kidney tissue was fixed in 4% paraformaldehyde for 3 days and dehydrated using an alcohol gradient. The tissue was then immersed in an ethanol–xylene (1:1) mixture in xylene for 15–20 min until the tissue became transparent. Transparent tissue was placed in a mixture of paraffin and xylene (1:1) for 1 h, followed by paraffin embedding. Paraffin sections were cut into 5-μm-thick sections and dried in a 37°C incubator. The immunohistochemistry kit was purchased from MXB Biotechnologies (KIT-9710 and DAB-0031, China). Renal tissue paraffin slices were washed with water and subjected to antigen retrieval. The slices were then incubated with primary antibodies against Kim-1 (Abcam, 1:200, ab78494), NGAL (Abcam, 1:200, ab70287), and the appropriate biotin-conjugated secondary immunoglobulin G (1:1000, S0001, Affinity, USA). After mounting the slices, co-stained images were captured using a Nikon Eclipse 90i fluorescence microscope.

### Immunofluorescence staining

Fluorescein-labeled antibodies were used against the corresponding antigens. PBS (0.01 mol/L, pH 7.4) was replaced every 10 min to maintain specimen humidity. The specimens were covered with fluorescent-labeled antibodies and stored in an enamel box for 30 min. The glass slide held on the glass stand was rinsed with PBS solution (0.01 mol/L, pH 7.4) in three cylinders, each washed for 3–5 min under oscillation. Excess moisture was absorbed from the glass slide, and a drop of glycerol was added to cover the glass. The slides were observed under a fluorescence microscope. The primary perforin antibody was purchased from Thermo Fisher Scientific (1:500, #MA5-12469, USA), and secondary fluorescent immunoglobulin G was purchased from Abmart (1:1000, M21014S, China).

### Western blot analysis

Kidney tissues were lysed using radioimmunoprecipitation assay (RIPA) buffer containing a protease inhibitor cocktail (Sigma-Aldrich, PE0230, USA). Samples were vortexed and centrifuged at 15,000 × g, 4°C for 25 min. The whole-cell lysate was collected and separated using SDS-PAGE (100 V, 1.5 h), transferred to a PVDF membrane (20 V, 30 min), and blocked for 2 h. The membrane was then probed with primary antibodies for 1 h at 37°C and with the corresponding secondary antibody at room temperature for 30 min. Immunoreactive bands were detected using enhanced chemiluminescence and quantified using ImageJ software. Primary antibodies against STING (1:500; #13647), IRF3 (1:500; #4302), and P-IRF3 (1:500; #29047) were purchased from Cell Signaling Technology (USA). Anti-perforin antibody (1:500, #MA5-12469) was obtained from Thermo Fisher Scientific. Antibodies against Kim-1 (1:1000, ab233720) and NGAL (1:1000, ab63929, ab188551) were obtained from Abcam.

### qRT-PCR analysis

Total RNA was extracted from the kidneys using TRIzol and Zymogen RNA extraction kits (Takara, RR820A, China). cDNA was synthesized using the PrimeScript RT Reagent kit (Takara, RR047A, China) in accordance with the manufacturer’s instructions. Gene-specific primers (Life Technologies, Carlsbad, CA, USA) were designed, and quantitative real-time PCR was performed on an ABI 7500 system. A comparison threshold was used to calculate the absolute mRNA number. Quantitative PCR mRNA data were normalized to that of the *GAPDH* signal used as an internal control (Table 3). The normalized delta threshold cycle value was calculated according to the manufacturer’s instructions.

**Table 3:** Primer sequences used in real-time PCR analysis.

**Target gene**	**Forward primer (5′–3′)**	**Reverse primer (5′–3′)**
mus-*STING*	AGGAGGAGGTTACCATGAATG	ATACCACTGATGAGGAGTCTTG
mus-*Irf3*	TGTGATGGTCAAGGTTGTTCC	GATAGGCTGGCTGTTGGAGAT
mus-*Perforin*	CACAGTAGAGTGTCCGATGTA	CTTGGTTCCCGAAGGAGCAGAT
mus -*IFN-γ*	ATATCTGGAGGAACTGGCAAA	GGTGTGATTCAATGACGCTTAT
mus-*GAPDH*	TGTGTCCGTCGTGGATCTGA	TTGCTGTTGAAGTCGCAGGAG
homo-*STING*	CGAACTTACAATCAGCATTACAA	CAGCCATACTCAGGTTATCAG
homo-*Irf3*	TAAGCCAGACCTGCCAACCTG	GGTCCTCTGCTAAACGCAAC
homo-*Perforin*	AGTGCCGCTTCTACAGTTTC	GGTGCCGTAGTTGGAGATAAG
homo-*IFN-γ*	TCGGTAACTGACTTGAATGT	TTACTGGGATGCTCTTCG
homo-*Kim-1*	GACAGAGTCTTCAGATGGCCT	GAGCAAGAAGCACCAAGACAG
homo-*NGAL*	TTGGGACAGGGAAGACGA	TCACGCTGGGCAACATTA
homo-*GAPDH*	GCACCGTCAAGGCTGAGAAC	ATGGTGGTGAAGACGCCAGT

### Statistical analysis

Data are expressed as mean ± standard error (SE). Comparisons between time points were performed using one-way analysis of variance (ANOVA), followed by Tukey’s honest significant difference (HSD) test. Differences were considered statistically significant at *P* < 0.05.

### Data availability statement

The authors confirm that the data supporting the findings of this study are available within the article.

## RESULTS

### Transcriptome analysis of AKI revealed that AKI was affected by immune regulation

**Figure 2:**
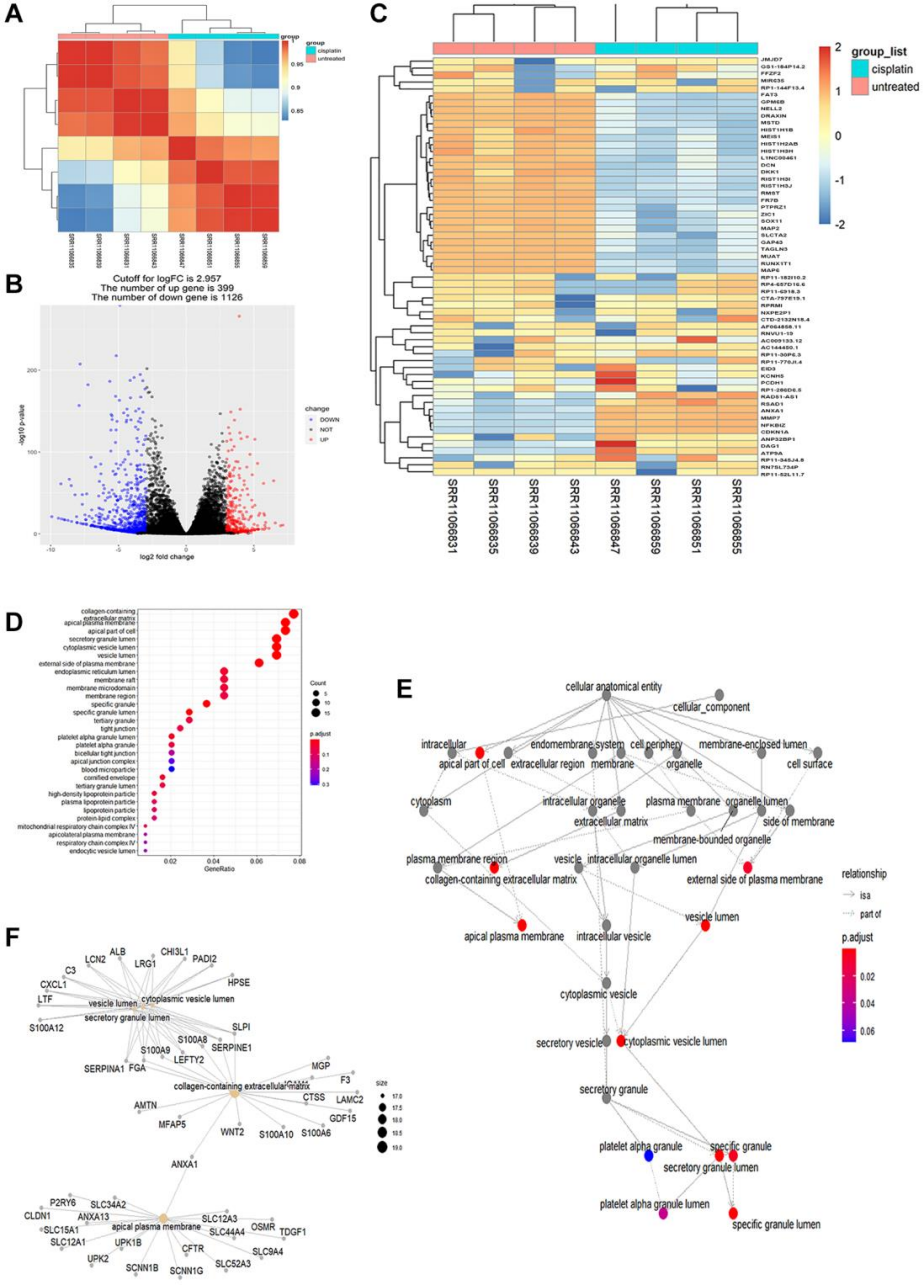
**Differential transcriptome analysis of AKI samples.** (**A**) Correlation of heatplot of differences between groups: a significant difference was observed between cisplatin and untreated groups in all the differently expressed genes (*P* < 0.05). (**B**) Differential gene volcano plot: 399 differentially expressed genes were up-regulated and 1126 differentially expressed genes were downregulated. The cutoff for logFC is 2.957. (**C**) Heatplot of the top 30 up-regulated genes and the top 30 downregulated genes. (**D**) Top 30 GO enriched analysis results in the dotplot (*P* < 0.05). (**E**) Correlation of the GO enriched analysis results in the goplot (*P* < 0.05). (**F**) Correlation of differently expressed genes in the GO enriched analysis in the cnetplot.

**Figure 3:**
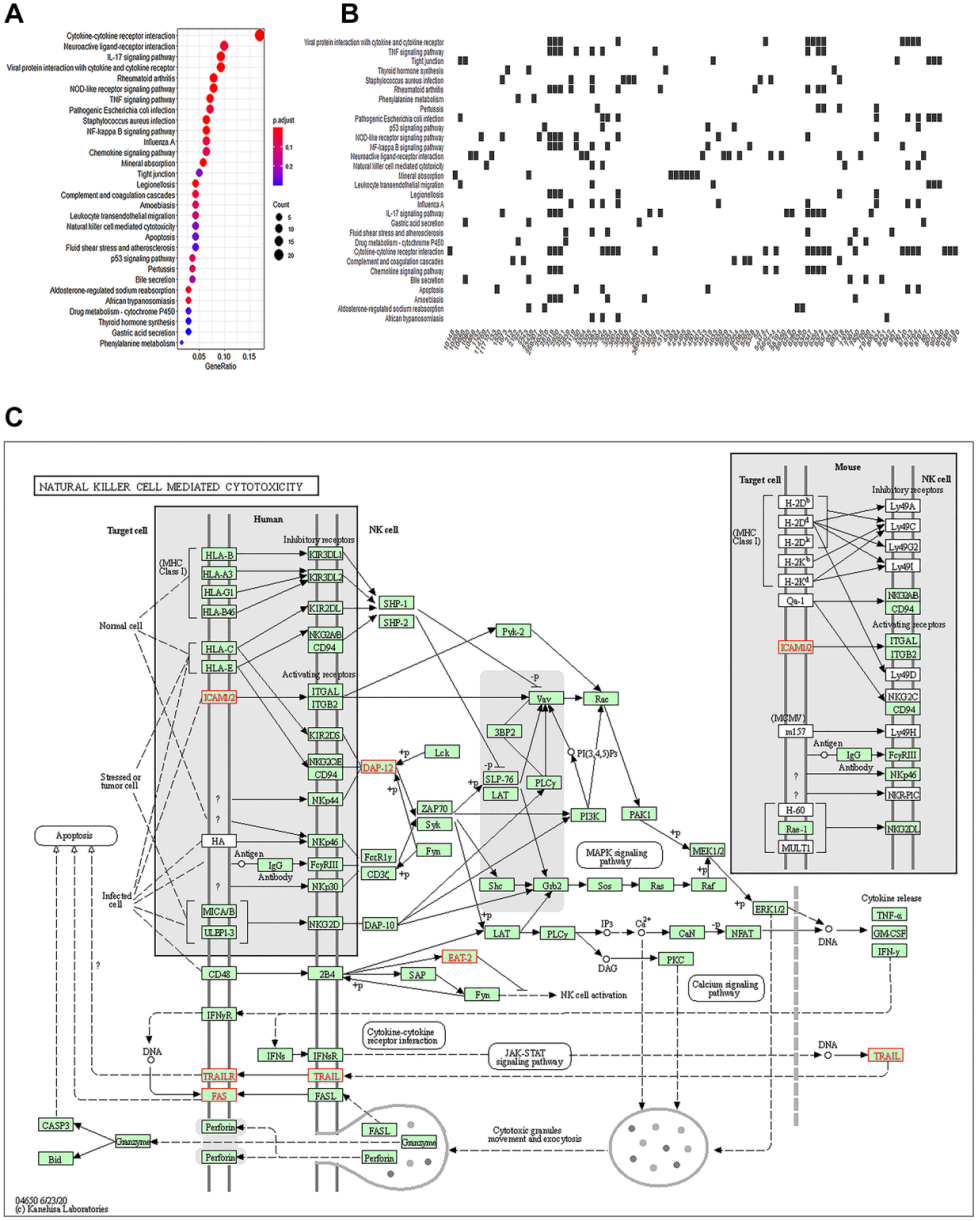
**Natural killer cells participate in immune regulation of AKI.** (**A**) Top 30 KEGG enriched analysis results in the dotplot (*P* < 0.05). (**B**) Heatplot of the regulated genes. (**C**) NK cell-mediated cytotoxicity pathways.

Quality control of the pruned gene sequence data revealed standard quality (Supplementary Figure 1). After comparing and enumerating the pruned gene data, the differentially expressed genes (DEGs) with statistical significance were screened using the Bioconductor package DESeq2 (*P* < 0.05) (Figure 2A). In the volcano plot, 399 DEGs were upregulated, and 1126 DEGs were downregulated (Figure 2B). A heat plot of the top 30 upregulated and top 30 downregulated genes was constructed (Figure 2C). The upregulated DEGs were selectively enriched by GO using *P* < 0.05 as screening criteria. Our data showed that GO cell components were mainly enriched in the secretory granule lumen, apical plasma membrane, cytoplasmic vesicle lumen, and vesicle lumen (Table 4, Figure 2D–2F). The KEGG analysis showed enrichment of cytokines and cytokine receptors, inflammatory factors, tumor necrosis factor, apoptosis, and other pathways (Table 5, Figure 3A, 3B). Notably, NK cell-mediated cytotoxicity pathways were also enriched. However, the association between AKI and NK cell-mediated cytotoxicity has rarely been investigated. The correlation between NK cell-mediated signaling and AKI requires further investigation (Figure 3C).

**Table 4:** Top 15 enriched results in GO analysis.

**ID**	**Description**	**GeneRatio**	**BgRatio**
GO:0034774	Secretory granule lumen	17/246	322/19559
GO:0016324	Apical plasma membrane	18/246	361/19559
GO:0060205	Cytoplasmic vesicle lumen	17/246	326/19559
GO:0031983	Vesicle lumen	17/246	328/19559
GO:0062023	Collagen-containing extracellular matrix	19/246	427/19559
GO:0045177	Apical part of cell	18/246	433/19559
GO:0035580	Specific granule lumen	7/246	62/19559
GO:0042581	Specific granule	9/246	160/19559
GO:0009897	External side of plasma membrane	15/246	417/19559
GO:0031093	Platelet alpha granule lumen	5/246	67/19559
GO:0005788	Endoplasmic reticulum lumen	11/246	308/19559
GO:0001533	Cornified envelope	4/246	45/19559
GO:0045121	Membrane raft	11/246	329/19559
GO:0098857	Membrane microdomain	11/246	330/19559
GO:0098589	Membrane region	11/246	343/19559

**Table 5:** Top 30 enriched analysis results in KEGG.

**ID**	**Description**	**GeneRatio**	**BgRatio**
hsa04060	Cytokine-cytokine receptor interaction	24/140	295/8102
hsa04657	IL-17 signaling pathway	13/140	94/8102
hsa04061	Viral protein interaction with cytokine and cytokine receptor	13/140	100/8102
hsa05323	Rheumatoid arthritis	11/140	93/8102
hsa04978	Mineral absorption	8/140	59/8102
hsa04668	TNF signaling pathway	10/140	112/8102
hsa05150	Staphylococcus aureus infection	9/140	96/8102
hsa04064	NF-kB signaling pathway	9/140	104/8102
hsa04621	NOD-like receptor signaling pathway	11/140	181/8102
hsa05134	Legionellosis	6/140	57/8102
hsa05130	Pathogenic *Escherichia coli* infection	10/140	197/8102
hsa04080	Neuroactive ligand-receptor interaction	14/140	341/8102
hsa05164	Influenza A	9/140	172/8102
hsa04610	Complement and coagulation cascades	6/140	85/8102
hsa04960	Aldosterone-regulated sodium reabsorption	4/140	37/8102
hsa05143	African trypanosomiasis	4/140	37/8102
hsa04062	Chemokine signaling pathway	9/140	192/8102
hsa05146	Amoebiasis	6/140	102/8102
hsa04115	p53 signaling pathway	5/140	73/8102
hsa05133	Pertussis	5/140	76/8102
hsa04670	Leukocyte transendothelial migration	6/140	114/8102
hsa04976	Bile secretion	5/140	90/8102
hsa04650	Natural killer cell mediated cytotoxicity	6/140	131/8102
hsa04530	Tight junction	7/140	169/8102
hsa04210	Apoptosis	6/140	136/8102
hsa05418	Fluid shear stress and atherosclerosis	6/140	139/8102
hsa00360	Phenylalanine metabolism	2/140	17/8102
hsa00982	Drug metabolism-cytochrome P450	4/140	72/8102
hsa04918	Thyroid hormone synthesis	4/140	75/8102

### 2’-deoxyadenosine administration attenuated tubular injury in AKI mice induced by FA

**Figure 4:**
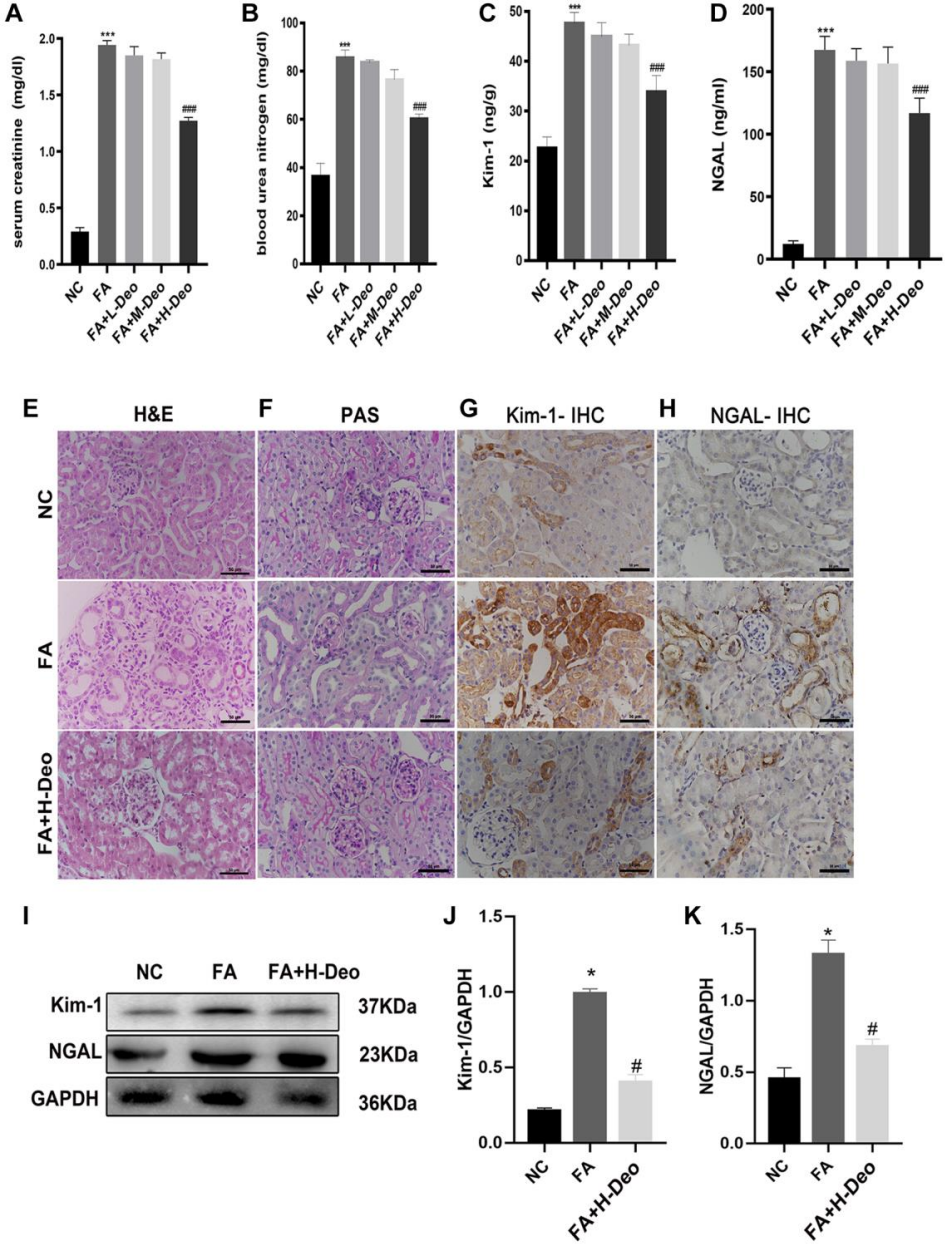
**2’-deoxyadenosine administration attenuated the development of FA-induced AKI in mice.** (**A**, **B**) Renal function of all mice was assessed by SCr and BUN. (**C**, **D**) Kim-1 and NGAL results of the mice were measured by ELISA. (**E**, **F**) Representative H&E and PAS of renal tissues obtained from specimens in different group. (**G**, **H**) Expression of Kim-1 and NGAL was determined by IHC. (**I**–**K**) Protein levels of NGAL and Kim-1 protein expression in the kidney were evaluated by western blot analysis. ^*^P indicates a significant difference between the NC group and the FA group. ^#^*P* represents the difference between the FA+Deo group and the FA group. *P* < 0.05. Values are shown as means ± SD from three independent experiments. Representative images are shown from a total of six animals per group.

To explore whether 2’-deoxyadenosine can reduce the injury level by regulating the immune response *in vivo*, we established a mouse model of AKI induced by FA. We treated it with different doses of 2’-deoxyadenosine. Two days after FA administration, the average SCr and BUN levels were significantly higher in the FA group than in the NC group. The SCr and BUN levels in the FA+H+Deo group were significantly lower than those in the FA group (Figure 4A, 4B). Similar features were observed for NGAL and Kim-1 in the FA-induced AKI mouse model (Figure 4C, 4D). Therefore, we selected high doses of 2’-deoxyadenosine for subsequent experiments.

To illustrate the therapeutic effect of high-dose 2’-deoxyadenosine in AKI mice, we stained kidney samples from mice with H&E and PAS. Morphological changes in FA-induced AKI mice primarily manifested in the proximal tubules of the subcapsular region of the renal cortex. Obvious edema and vacuoles in the renal proximal tubule epithelial cells dilated the renal tubule lumen, and swelling of the mesangial spaces and cells in some glomeruli suggested kidney damage. The specimens in the FA+H-Deo group exhibited milder pathological damage than those in the FA group, suggesting that the substantial damage to the kidney caused by FA injection was markedly prevented with the high doses of 2’-deoxyadenosine treatment (Figure 4E, 4F). IHC staining of the specimens confirmed that the AKI markers NGAL and Kim-1 expression levels were remarkably higher in the AKI group. This upregulation was blocked by high doses of 2’-deoxyadenosine treatment (Figure 4G, 4H). The western blotting analysis consistently indicated the preventive function of high doses of 2’-deoxyadenosine treatment against AKI (Figure 4I–4K).

### 2’-deoxyadenosine reduced perforin expression through the STING/IRF3 signaling pathway in the FA-induced AKI mouse model

**Figure 5:**
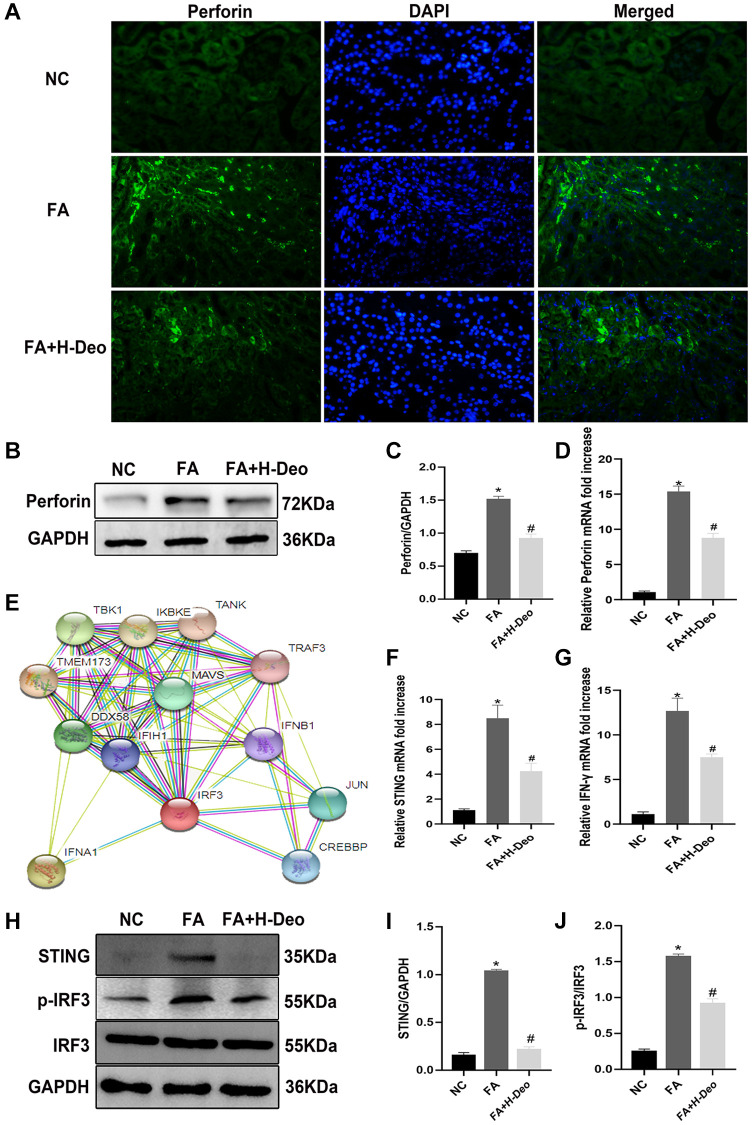
**2’-deoxyadenosine protected against AKI by inhibiting perforin expression in NK cells by regulating STING/IRF3 signaling pathway *in vivo*.** (**A**) Immunofluorescence studies of perforin expression on NK cells after FA treatment. DAPI staining was used to determine the number of nuclei. Anti-perforin staining was used to determine perforin expression (×400). (**B**, **C**) Protein levels of perforin in the kidney were evaluated by western blot analysis. (**D**) The mRNA levels of perforin in the kidney were evaluated by qRT-PCR analysis. (**E**) Bioinformatics of STING (TMEM173) and its regulated IRF3 from STRING PPI database. (**F**, **G**) The mRNA levels of STING and IFN-γ in kidney were evaluated by qRT-PCR analysis. (**H**–**J**) Protein levels of STING, p-IRF3/IRF3 expression in kidney were evaluated by western blot analysis. ^*^P indicates a significant difference between the NC group and the FA group. ^#^*P* represents the difference between the FA+Deo group and the FA group. *P* < 0.05. Values are shown as means ± SD from three independent experiments. Representative images are shown from a total of six animals per group.

Perforin plays a major role in NK cell-mediated TEC injury. NK cell infiltration in kidney tissues was detected by immunofluorescence staining for the NK cell marker perforin (Figure 5A). These observations were further confirmed at the protein and mRNA levels by WB and qRT-PCR, respectively. FA mice showed significantly increased perforin expression compared with the NC group. These effects were alleviated by high doses of 2’-deoxyadenosine treatment (Figure 5B–5D). Bioinformatic analysis of PPI revealed an interaction between STING (TMEM173) and its downstream signaling molecules, TBK1, IRF3, and type I IFN (Figure 5E). IFN-γ (type II IFN), an important inflammatory indicator of kidney injury, was mainly released from NK and NKT cells. Through qRT-PCR analyses, we observed high expression of STING in AKI mice, and found that high doses of 2’-deoxyadenosine can reduce the expression level (Figure 5F). Treatment can also effectively reduce the release of inflammatory factors IFN-γ released by NK cells (Figure 5G). To verify the *in vivo* results of the inhibitory effect of high doses of 2’-deoxyadenosine on the STING pathway, we evaluated the levels of STING and IRF3 phosphorylation in kidney homogenates and found that they were downregulated upon high doses of 2’-deoxyadenosine injections by western blotting (Figure 5H–5J). These results confirmed the suppressive effect of high doses of 2’-deoxyadenosine on perforin and IFN-γ release via inhibiting STING/TBK1/IRF3 expression.

### 2’-deoxyadenosine protects against AKI by inhibiting perforin expression in NK cells via the STING/IRF3 pathway *in vitro*

**Figure 6:**
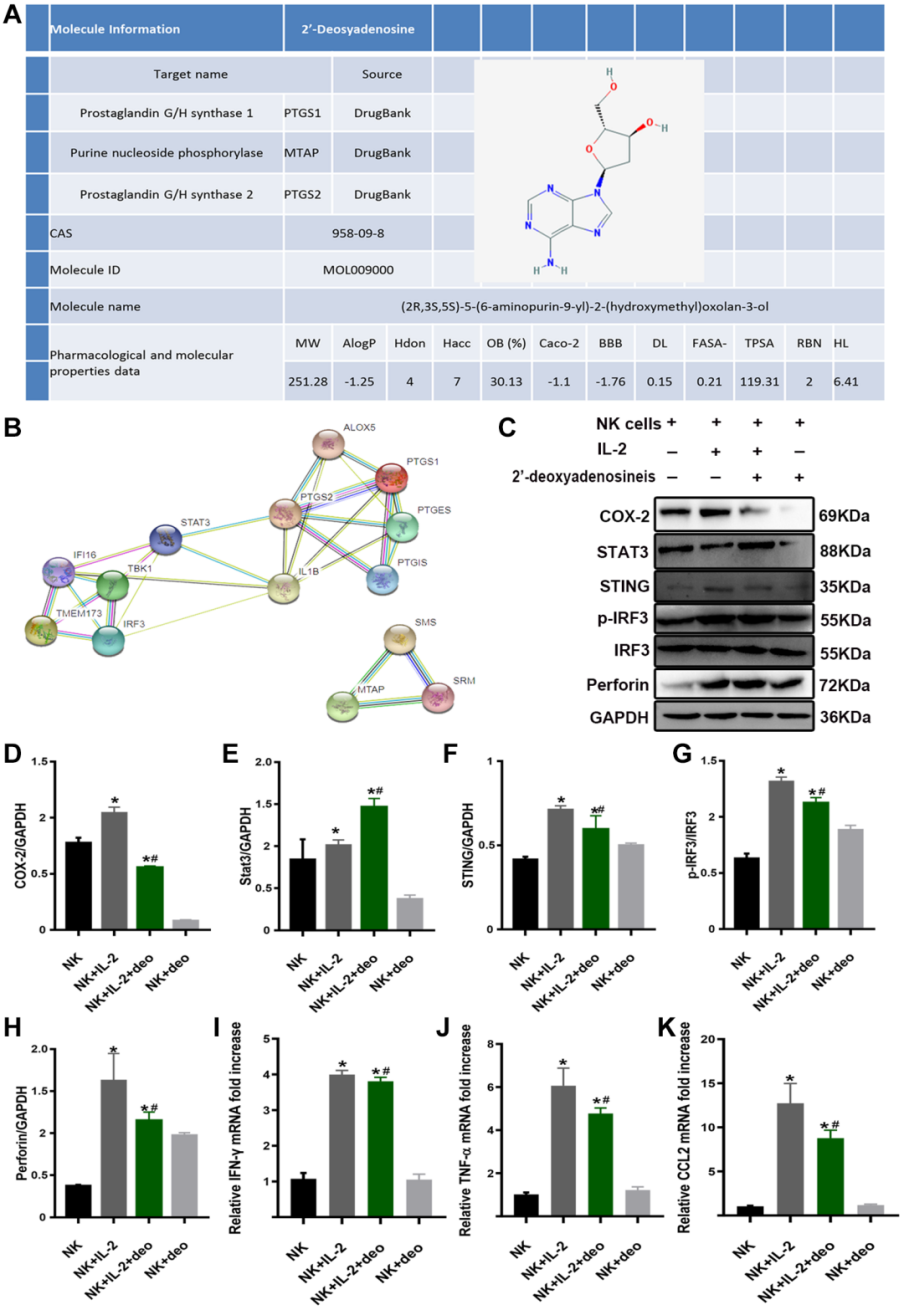
**2’-deoxyadenosine inhibited the activity of NK cells by regulating the STING/IRF3 signaling pathway *in vitro*.** (**A**) Related target of 2’-deoxyadenosine with PTGS1 (COX-1), PTGS2 (COX-2), and MTAP based on DrugBank and TCMSP analysis. (**B**) Bioinformatics of PTGS1 (COX-1), PTGS2 (COX-2), STAT3 and its regulated STING/IRF3 from STRING PPI database. (**C**–**H**) The protein levels of COX-2, STAT3, STING, IRF3, and perforin in NK cells were evaluated by western blot analysis. (NK: naive NK cells. NK+IL-2: NK cells+IL-2 group. NK+IL-2+deo: NK cells+IL-2+2’-deoxyadenosine group. NK+deo: naive NK cells+2’-deoxyadenosine group). (**I**–**K**) The mRNAs levels of IFN-γ, TNF-α and CCL2 expression in NK cells were evaluated by qRT-PCR analysis. (NK: naive NK cells. NK+IL-2: NK cells+IL-2 group. NK+IL-2+deo: NK cells+IL-2+2’-deoxyadenosine group. NK+deo: naive NK cells+2’-deoxyadenosine group.) ^*^P indicates a significant difference compared with the NK group. ^#^*P* represents the difference compared with the NK+IL-2 group. *P* < 0.05. Values are shown as means ± SD from three independent experiments.

To confirm the mechanism by which high doses of 2’-deoxyadenosine inhibited perforin and IFN-γ expressions in NK cells via STING/IRF3 signaling to attenuate AKI, we performed *in vitro* experiments. TCMSP analysis demonstrated that the composition of CS was consistent with the purification results. Based on the DrugBank database, the related targets of 2’-deoxyadenosine were identified as PTGS1 (COX-1), PTGS2 (COX-2), and MTAP (Figure 6A). PPI analysis revealed a strong correlation between PTGS1 (COX-1), PTGS2 (COX-2), STAT3, STING, TBK1, and IRF3 (Figure 6B). Furthermore, we explored whether 2’-deoxyadenosine could inhibit NK cell activation by inhibiting STING and IRF3 by targeting the inhibition of COX-2 and downstream STAT3.

Since IL-2 secreted by T lymphocytes is responsible for stimulating the growth and differentiation of T cells, B cells, and NK cells [32], we used 1000 IU/mL IL-2 to treat NK cells *in vitro* to determine the source of perforin and IFN-γ release. Strikingly, activation of NK cells resulted in significantly increased expressions of COX-2, STING, p-IRF3, and perforin and decreased STAT3 by western blotting, whereas concurrent exposure to 2’-deoxyadenosine inhibited these protein expressions and elevated STAT3 expressions (Figure 6C–6H). Next, we examined the mRNA expression of inflammatory factors by qRT-PCR. As indicated in Figure 6I–K, the mRNA levels of IFN-γ, TNF-α, and CCL2 (chemokine monocyte chemoattractant protein-1, MCP-1/CCL2) were significantly elevated in IL-2-treated NK cells compared with those in the naïve NK group and were remarkably relieved by treatment with 2’-deoxyadenosine.

## DISCUSSION

Drug-induced nephrotoxicity is a relatively common complication, occurring in 20% of patients with AKI. FA-induced AKI is a typical representative of nephrotoxic AKI and is a generally accepted model for studying the mechanisms underlying AKI [29, 33]. Body damage caused by FA is limited to the kidneys, thus eliminating mixed extra-renal factors. Local inflammatory responses triggered by FA administration in the kidney reflect upstream signals associated with inflammatory diseases, which are essential for tissue remodeling observed in many other forms of organ injury [34]. AKI induced by FA and other drugs, such as cisplatin, simulates important aspects of nephrotoxic AKI, including renal tubular epithelial cell injury, apoptosis, inflammatory cell infiltration, and immune system activation. Previous studies have demonstrated that kidney-infiltrating macrophages and neutrophils significantly increase after injury, indicating that innate immunity plays a major role in renal tubular dysfunction [35]. Bioinformatics analysis revealed that AKI-related immunomodulatory DEGs were enriched in NK cell-mediated cytotoxicity pathways. The function of NK cells in AKI progression has rarely been studied; thus, understanding the new trends in renal regenerative medicine and the correlation between AKI and NK cell signaling is imperative.

IFN-γ and TNF-α produced by NK cells form essential components of the innate immune response [36]. Zhang et al. showed that NK cells can kill TECs *in vitro* and that perforin plays a crucial role in the cytotoxic function of NK cells, promoting the development of kidney ischemia–reperfusion injury (IRI) [37]. NK cell activation occurs through specific pathways. However, the involvement of the STING/IRF3 pathway in NK cell-mediated TEC killing has not yet been confirmed.

STING is an essential signaling adaptor that links cytosolic DNA to the TBK1/IRF3 signaling axis, which induces a STING-dependent type I IFN response [38]. STING is an innate immune sensor consisting of cyclic dinucleotides [39]. Bioinformatics analysis revealed an interaction between STING and IRF3 (Figure 5E). It has been indicated that cGAMP and STING can activate NK cells [9, 40, 41]. IRF3 is responsible for the expression of several chemokines, including Rantes and IP-10, in NK cells. Activated NK cells express receptors for these chemokines [42].

CS has gained increasing attention recently owing to its renoprotective effects [43–45]. However, the mechanism by which CS protects against AKI, particularly the active components of CS, requires further investigation. In this study, we verified that the main active ingredient of CS treatment significantly improved renal function as assessed by the reduction of SCr and BUN levels, morphological amelioration at 48 h after FA stimulation, and alleviation of NGAL and Kim-1 deposition, which are biomarkers for early-stage AKI diagnosis [46–49]. Further, we isolated and predicted the targeting proteins of CS extract 2’-deoxyadenosine, including PTGS1 (COX-1) and PTGS2 (COX-2) (Figure 6A), which were key mediators of the inflammatory response. Because COX-2 is induced by inflammatory stimuli, it has traditionally been considered the most suitable target for anti-inflammatory drugs. Compared with COX-2^+/+^ mice, phosphorylated STAT3 protein levels increased significantly in COX-2^−/−^ mice after inflammatory stimulation. Activation of the STING pathway is essential for the sensitizing effect of STAT3 inhibition on STING signaling [50, 51]. Other studies have confirmed that CS can suppress the expression of COX-2 [52, 53]. Strikingly, we found that 2’-deoxyadenosine inhibited COX-2 expression and promoted STAT3 expression, which in turn downregulated the protein expression of STING/p-IRF3/perforin signaling (Figure 6C–6H). These results demonstrated that 2’-deoxyadenosine treatment alleviated NK cell activation through the COX-2/STAT3/STING/IRF3 pathway. Collectively, these data suggest that perforin plays a central role in NK cell-mediated kidney injury *in vivo* and NK cell damage *in vitro*. Further investigation of the potential functions of NK cells in AKI and the relevant regulatory network requires the development of therapeutic agents that specifically target NK cell activation signaling pathways.

## CONCLUSION

CS extract 2’-deoxyadenosine alleviated AKI by improving renal pathophysiological changes and inhibited the expression of perforin and IFN-γ released from NK cells via the STING/IRF3 signaling pathway, thereby reducing the damage to renal tubular epithelial cells. This study highlights the previously unrecognized role of NK cells in FA-induced AKI, which may lead to novel and clinically useful approaches for improving renal injuries induced by various causes of kidney inflammation, including nephrotoxic AKI, renal IRI, and transplantation.

## Supplementary Materials

Supplementary Figure 1
