# Supplementary figures and images for: Cordyceps sinensis extract protects against acute kidney injury by inhibiting perforin expression in NK cells via the STING/IRF3 pathway
Source: Aging (Albany NY). 2024 Mar 21;16(7):5887–904. doi: 10.18632/aging.205676 (PMC11042953; doi:10.18632/aging.205676)

SUPPLEMENTARY FIGURE

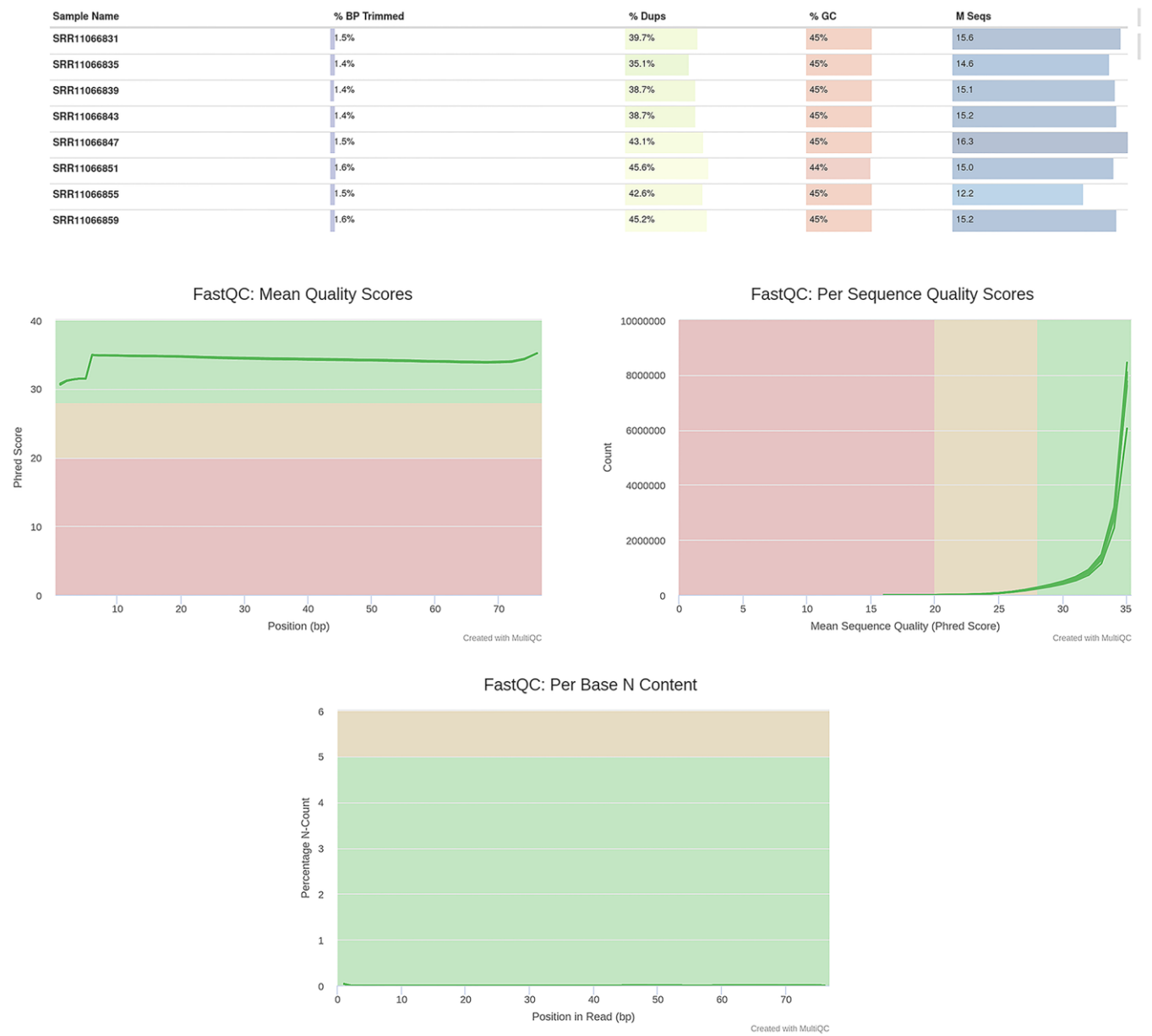

Supplementary Figure 1. Gene sequence quality control report.

Supplement: Supplementary Figure 1 [file aging-16-205676-s001.pdf]
